# Supplementary material for: Contribution of glutamatergic projections to neurons in the nonhuman primate lateral substantia nigra pars reticulata for the reactive inhibition
Source: bioRxiv. 2024 Dec 25:2024.12.25.630331. Preprint. [Version 1] doi: 10.1101/2024.12.25.630331 (PMC11703221; doi:10.1101/2024.12.25.630331)
Supplement: 1 [file NIHPP2024.12.25.630331V1-supplement-1.pdf]

Supplementary Methods

Detailed model specifications

Model specifications for the statistical analyses presented in the main text are detailed below. All linear mixed-effects (LMM) and generalized linear mixed-effects (GLMM) models included random effects (e.g., [1|monkey_ID] or [1|monkey_ID: Neuron_ID]) to statistically control for individual differences at both the subject and neuron levels. By accounting for these hierarchical structures, the models minimized the risk of inflated type I errors due to the nonindependence of observations within the same monkey or neuron, ensuring more accurate and robust inferences. All models were fit using RStudio with the following packages: lme4 (Bates et al., 2014), pbkrtest (Halekoh & Højsgaard, 2014), emmeans (Lenth et al., 2019), and brms (Bürkner, 2017).

1. Linear mixed-effects model for neuronal activity at scene onset (Figure 2D)

Model objective: To evaluate differences in neuronal activity following scene onset.

Model specifications:
Full Model: NormalizedNeuronalActivity ~ Scene + (1|monkey_ID) + (1|monkey_ID: Neuron_ID)
Null Model: NormalizedNeuronalActivity ~ (1|monkey_ID) + (1|monkey_ID: Neuron_ID)

Variables:
NormalizedNeuronalActivity: Mean Z-transformed PSTH (100-300 ms after scene onset)
Scene: Fixed effect (levels: 1-4)
monkey_ID, Neuron_ID: Random effects

Statistical threshold: $\alpha = 0.05$

2. Linear mixed-effects model for neuronal activity at target onset (Figure 2F)

bioRxiv preprint doi: https://doi.org/10.1101/2024.12.25.630331; this version posted December 25, 2024. The copyright holder for this preprint (which was not certified by peer review) is the author/funder. This article is a US Government work. It is not subject to copyright under 17 USC 105 and is also made available for use under a CC0 license.

Model objective: To examine how neuronal activity varies with scene context, object value, and target direction.

Model specifications:
Full Model: NormalizedNeuronalActivity ~ Scene × Value × Direction + (1|monkey_ID) + (1|monkey_ID: Neuron_ID)
Null Model: NormalizedNeuronalActivity ~ (1|monkey_ID) + (1|monkey_ID: Neuron_ID)

Variables:
NormalizedNeuronalActivity: Mean Z-transformed PSTH (100-300 ms after target onset)
Scene: Fixed effect (levels: 1-4)
Value: Fixed effect (levels: good, bad)
Direction: Fixed effect (levels: contralateral, ipsilateral)
monkey_ID, Neuron_ID: Random effects

Multiple comparisons:
6 pairwise comparisons (good vs. bad, contralateral vs. ipsilateral)
Bonferroni-corrected threshold: $\alpha = 0.05/6$

3. Linear mixed-effects model for neuronal activity at saccade onset (Figure S2C)
Model objective: To examine neuronal activity patterns aligned to saccade onset.

Model specifications:
Full Model: NormalizedNeuronalActivity ~ Scene × Value × Direction + (1|monkey_ID) + (1|monkey_ID: Neuron_ID)
Null Model: NormalizedNeuronalActivity ~ (1|monkey_ID) + (1|monkey_ID: Neuron_ID)

Variables:
NormalizedNeuronalActivity: Mean Z-transformed PSTH (from 150 ms before to 50 ms after saccade onset)
Scene: Fixed effect (levels: 1-4)
Value: Fixed effect (levels: good, bad)
Direction: Fixed effect (levels: contralateral, ipsilateral)
monkey_ID, Neuron_ID: Random effects

Multiple comparisons:
6 pairwise comparisons (good vs. bad, contralateral vs. ipsilateral)
Bonferroni-corrected threshold: $\alpha = 0.05/6$

4. Bayesian linear mixed-effects model for neural activity and reaction time (Figure S2D)
Model objective: To examine the relationship between neural activity and saccadic reaction times.

Model specifications:
NormalizedRT ~ NormalizedNeuronalActivity + (1|monkey_ID) + (1|monkey_ID: Neuron_ID)

Variables:
NormalizedRT: Standardized reaction times (mean = 0, SD = 1)
NormalizedNeuronalActivity: Mean Z-transformed PSTH (from 150 ms before to 50 ms after saccade onset)
monkey_ID, Neuron_ID: Random effects

MCMC specifications:
Chains: 4
Iterations: 50,000 per chain
Warmup: 5,000 per chain

bioRxiv preprint doi: https://doi.org/10.1101/2024.12.25.630331; this version posted December 25, 2024. The copyright holder for this preprint (which was not certified by peer review) is the author/funder. This article is a US Government work. It is not subject to copyright under 17 USC 105 and is also made available for use under a CC0 license.

Convergence criteria: Rhat = 1.00
These parameters (number of chains, total iterations, and warmup period) were selected to ensure adequate, effective sample sizes, stable parameter estimates, and reliable convergence diagnostics, thereby enhancing the credibility of our posterior inferences.

Initial model fitting using mean RT values failed to converge. As a result, RTs were standardized (mean = 0, SD = 1) prior to model estimation.
Model convergence was evaluated using the potential scale reduction factor (Rhat) and effective sample sizes (ESS). All Rhat values were 1.00, indicating successful convergence. Posterior distributions were summarized by their mean, 95% credible intervals (CIs), and probabilities that the regression coefficient for NormalizedFR (β_1) was greater than 0. Statistical significance was inferred if the 95% CI did not include 0 and the probability of a positive coefficient exceeded 95%.
All analyses were performed separately for four conditions: contralateral good objects, contralateral bad objects, ipsilateral good objects, and ipsilateral bad objects.

5. Linear mixed-effects model for neuronal activity during choice rejection and fixation (Figures 3E and H)
Model objective: To compare neuronal activity patterns between different rejection strategies and across choice and fixation tasks.

Model specifications:
Full Model: NormalizedNeuronalActivity ~ Condition × Direction + (1|monkey_ID) + (1|monkey_ID: Neuron_ID)
Null Model: NormalizedNeuronalActivity ~ (1|monkey_ID) + (1|monkey_ID: Neuron_ID)

Variables:
NormalizedNeuronalActivity: Mean Z-transformed PSTH (100-300 ms post-target)
Condition: Fixed effect (levels: return (choice task), stay (choice task), good (fixation task), bad (fixation task))
Direction: Fixed effect (levels: contralateral, ipsilateral)

Multiple comparisons:
6 pairwise comparisons between conditions
Bonferroni-corrected threshold: $\alpha = 0.05/6$

6. Generalized linear mixed-effects model for saccade reaction times after injection (Figure 4A)
Model objective: To investigate the effects of glutamatergic antagonist injection on saccadic reaction times.

Model specifications:
Full Model: MedianSaccadeReactionTimes ~ Injection × PrePost × Value × Direction + (1|monkey_ID) + (1|monkey_ID: Session_ID)
Null Model: MedianSaccadeReactionTimes ~ (1|monkey_ID) + (1|monkey_ID: Session_ID)

Variables:
MedianSaccadeReactionTimes: Median reaction time per condition
Injection: Fixed effect (levels: antagonist, saline)
PrePost: Fixed effect (levels: preinjection, postinjection)
Value: Fixed effect (levels: good, bad)
Direction: Fixed effect (levels: contralateral, ipsilateral)
Session_ID: Random effect identifying individual injection sessions

Distribution: Poisson
Rationale for distribution choice: Reaction times are non-negative count data characterized by a right-skewed distribution.

bioRxiv preprint doi: https://doi.org/10.1101/2024.12.25.630331; this version posted December 25, 2024. The copyright holder for this preprint (which was not certified by peer review) is the author/funder. This article is a US Government work. It is not subject to copyright under 17 USC 105 and is also made available for use under a CC0 license.

Multiple comparisons:
8 pairwise comparisons (pre vs. post for each condition)
Bonferroni-corrected threshold: α = 0.05/8

7. Generalized linear mixed-effects model for chosen action rate after injection (Figure 4B)
Model objective: To investigate the impact of glutamatergic antagonist injection on action selection for bad objects.

Model specifications:
Full Model: ChosenActionRate ~ Injection × PrePost × Value × Direction + (1|monkey_ID) + (1|monkey_ID: Session_ID), weights = (total trial count)
Null Model: ChosenActionRate ~ (1|monkey_ID) + (1|monkey_ID: Session_ID), weights = (total trial count)

Variables:
ChosenActionRate: Proportion of selected actions
Injection, PrePost, Value, Direction: Fixed effects as described above
total_trial_count: Weights to account for different numbers of trials

Distribution: Binomial
Rationale for distribution choice: Analysis of proportional data with binary outcomes

Multiple comparisons:
8 pairwise comparisons
Bonferroni-corrected threshold: α = 0.05/8

8. Generalized linear mixed-effects model for fixation break error rate after injection (Figure 4C)
Model objective: To examine how glutamatergic antagonist injection affects the ability to suppress reflexive saccades.

Model specifications:
Full Model: FixBreakErrorRate ~ Injection × PrePost × Value × Direction + (1|monkey_ID) + (1|monkey_ID: Session_ID), weights = (total trial count)
Null Model: FixBreakErrorRate ~ (1|monkey_ID) + (1|monkey_ID: Session_ID), weights = (total trial count)

Variables:
FixBreakErrorRate: Proportion of fixation break errors
All other variables, as defined above

Distribution: Binomial
Rationale for distribution choice: Analysis of error rate data with binary outcomes.

Multiple comparisons:
8 pairwise comparisons
Bonferroni-corrected threshold: α = 0.05/8

bioRxiv preprint doi: https://doi.org/10.1101/2024.12.25.630331; this version posted December 25, 2024. The copyright holder for this preprint (which was not certified by peer review) is the author/funder. This article is a US Government work. It is not subject to copyright under 17 USC 105 and is also made available for use under a CC0 license.

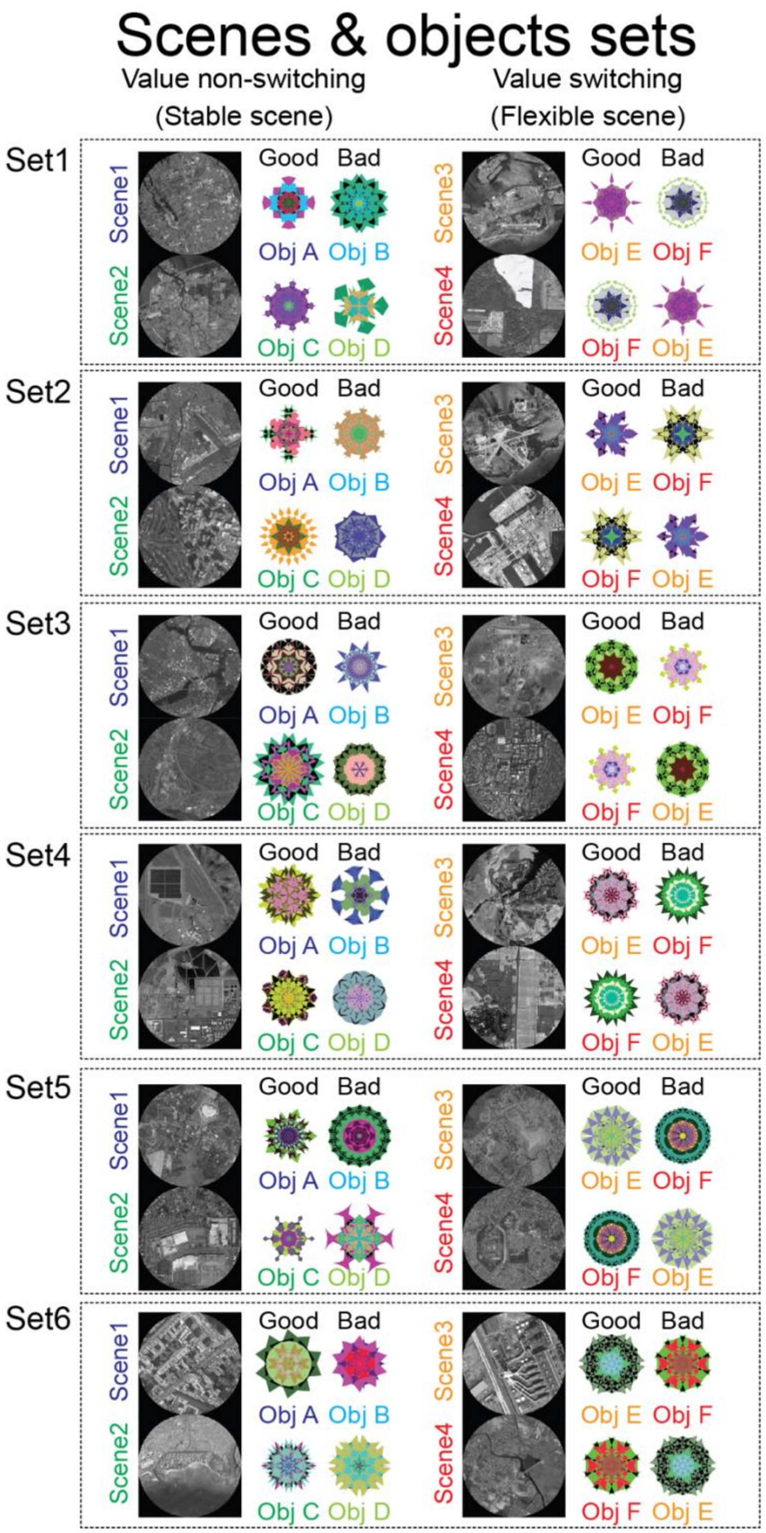

Fig. S1. All sets of scenes 1-4 and good and bad objects for the choice task.

bioRxiv preprint doi: https://doi.org/10.1101/2024.12.25.630331; this version posted December 25, 2024. The copyright holder for this preprint (which was not certified by peer review) is the author/funder. This article is a US Government work. It is not subject to copyright under 17 USC 105 and is also made available for use under a CC0 license.

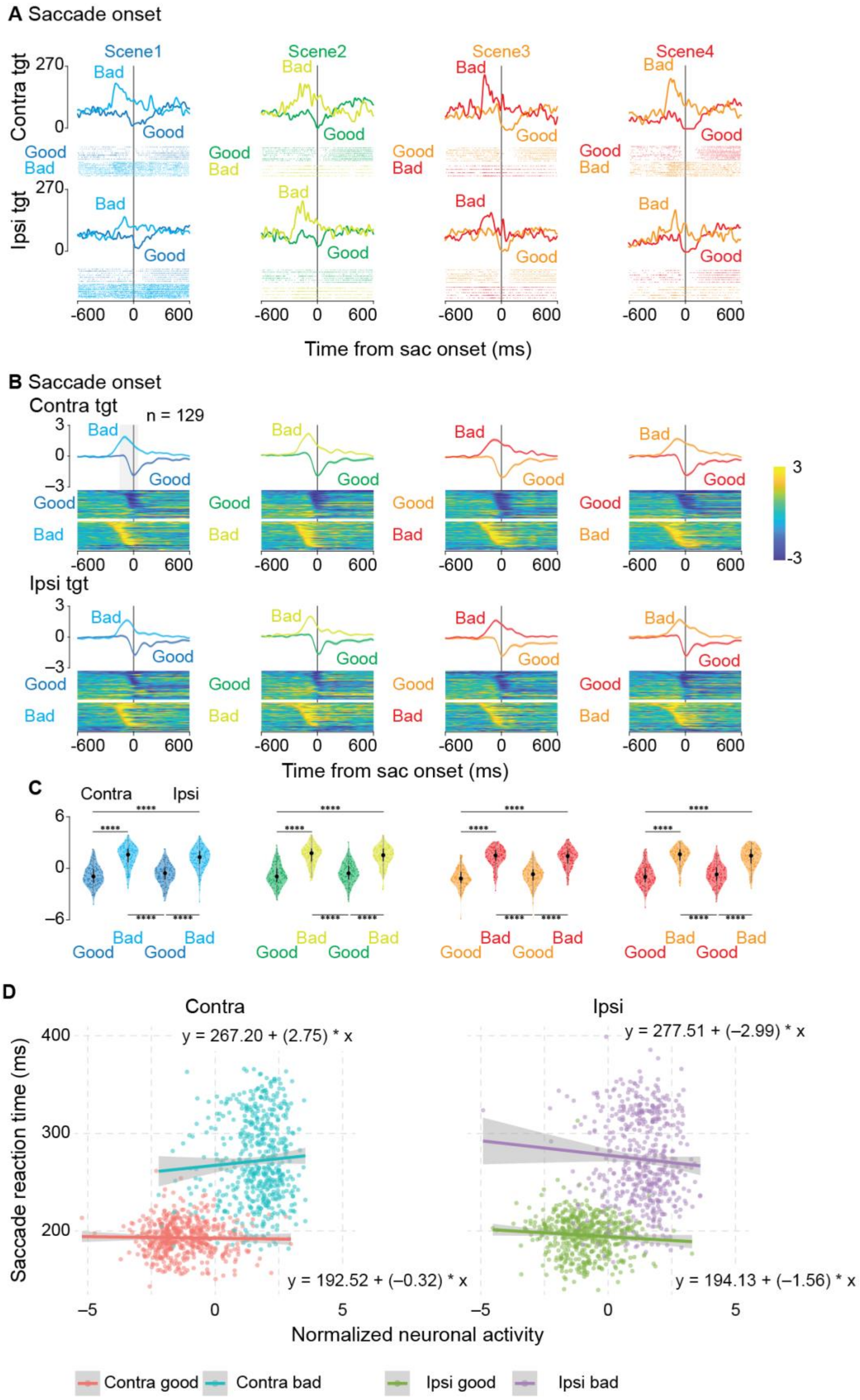

bioRxiv preprint doi: https://doi.org/10.1101/2024.12.25.630331; this version posted December 25, 2024. The copyright holder for this preprint (which was not certified by peer review) is the author/funder. This article is a US Government work. It is not subject to copyright under 17 USC 105 and is also made available for use under a CC0 license.

Fig. S2. Neuronal activity aligned to saccade onset during the choice task

(A) Activity of the same representative SNr neuron shown in Figure 2B, now aligned to saccade onset. Data are displayed separately for contralateral (upper) and ipsilateral (lower) target presentations, with different colors representing responses to good and bad objects in each scene.

(B) Population activity aligned to saccade onset (n = 129 neurons). Upper panels display mean firing rates for contralateral target presentations, while lower panels show those for ipsilateral presentations. Population averages (upper traces) and normalized single-neuron responses (heat maps) are shown for good and bad objects across all scenes.

(C) Distribution of neuronal responses. Violin plots quantify activity during a 200-ms window spanning from 150 ms before to 50 ms after saccade onset. Asterisks denote significant differences between conditions (*p < 0.05, **p < 0.01, ***p < 0.001, ****p < 0.0001, post-hoc pairwise t-tests with Bonferroni correction).

(D) Relationship between normalized neuronal activity and saccadic reaction times for different target conditions. Scatter plots show individual trials with regression lines (colored lines) and 95% confidence intervals (gray shading). Separate analyses are presented for contralateral good (red), contralateral bad (cyan), ipsilateral good (green), and ipsilateral bad (purple) targets. Regression equations are provided for each condition.

bioRxiv preprint doi: https://doi.org/10.1101/2024.12.25.630331; this version posted December 25, 2024. The copyright holder for this preprint (which was not certified by peer review) is the author/funder. This article is a US Government work. It is not subject to copyright under 17 USC 105 and is also made available for use under a CC0 license.

Table S1. Saccade reaction times in each condition of each monkey.

**Monkey Ch**	n	mean RT (ms)	SD	95% CI
**Good obj**				
ObjA in Scene1	448	195.5	24.0	[193.3 197.8]
ObjC in Scene2	445	193.0	32.9	[190.0 196.1]
ObjE in Scene3	451	202.4	25.6	[200.0 204.7]
ObjF in Scene4	456	202.2	23.7	[200.0 204.4]
**Bad obj**				
ObjB in Scene1	406	252.6	29.9	[249.7 255.6]
ObjD in Scene2	430	244.4	40.7	[240.5 248.2]
ObjF in Scene3	410	229.7	40.2	[225.8 233.6]
ObjE in Scene4	481	229.6	41.6	[225.9 233.3]
**Monkey Cr**	n	mean RT (ms)	SD	95% CI
**Good obj**				
ObjA in Scene1	980	181.5	29.7	[179.7 183.4]
ObjC in Scene2	976	184.3	28.5	[182.5 186.1]
ObjE in Scene3	975	192.4	29.4	[190.6 194.3]
ObjF in Scene4	958	184.7	27.2	[183.0 186.5]
**Bad obj**				
ObjB in Scene1	852	246.3	48.0	[243.0 249.5]
ObjD in Scene2	782	259.2	46.2	[256.0 262.4]
ObjF in Scene3	865	236.4	46.7	[233.3 239.5]
ObjE in Scene4	887	241.4	48.6	[238.2 244.6]
**Monkey Sp**	n	mean RT (ms)	SD	95% CI
**Good obj**				
ObjA in Scene1	910	196.7	19.5	[194.4 197.0]
ObjC in Scene2	897	197.3	19.9	[196.0 198.6]
ObjE in Scene3	897	204.4	20.1	[203.1 205.6]
ObjF in Scene4	883	203.3	22.3	[201.8 204.8]
**Bad obj**				
ObjB in Scene1	585	321.4	40.0	[318.2 324.7]
ObjD in Scene2	561	325.3	41.4	[321.8 328.7]
ObjF in Scene3	698	309.4	47.2	[305.9 313.0]
ObjE in Scene4	724	315.6	48.5	[312.1 319.1]

bioRxiv preprint doi: https://doi.org/10.1101/2024.12.25.630331; this version posted December 25, 2024. The copyright holder for this preprint (which was not certified by peer review) is the author/funder. This article is a US Government work. It is not subject to copyright under 17 USC 105 and is also made available for use under a CC0 license.

Table S2. Counts of chosen actions for Bad objects

**Monkey Ch**	total	accept	Return	Stay	other	fxbreak
Scene1	545	0	412	92	17	24
Scene2	502	0	424	51	9	18
Scene3	469	0	410	41	1	17
Scene4	547	0	462	63	7	15
non-switch(scene1,2)	1044	0	836	143	26	42
switch(scene3,4)	1016	0	872	104	8	32
**Monkey Cr**	total	accept	Return	Stay	other	fxbreak
Scene1	1050	1	872	156	1	20
Scene2	996	2	802	159	1	32
Scene3	978	15	903	50	1	9
Scene4	1051	14	902	110	2	23
non-switch(scene1,2)	2046	3	1674	314	2	52
switch(scene3,4)	2029	29	1805	160	3	32
**Monkey Sp**	total	accept	Return	Stay	other	fxbreak
Scene1	866	2	581	278	0	5
Scene2	842	1	557	282	0	2
Scene3	923	4	692	225	0	2
Scene4	946	2	729	213	0	2
non-switch(scene1,2)	1708	3	1138	560	0	7
switch(scene3,4)	1869	6	1421	438	0	4

bioRxiv preprint doi: https://doi.org/10.1101/2024.12.25.630331; this version posted December 25, 2024. The copyright holder for this preprint (which was not certified by peer review) is the author/funder. This article is a US Government work. It is not subject to copyright under 17 USC 105 and is also made available for use under a CC0 license.

Table S3. Summary of statistical test to compare the normalized neuronal activity of SNr neurons at target onset among conditions during choice task in Figure 2.

SNr						
parametric bootstrap test (n = 10,000)	*p*					
full model vs. null model	< .0001					
post hoc (pairwise t-test, Bonferroni correction)	Mean (SD)	Mean (SD)	*t*	*p*	95% CI	effect size
**Scene1**						
(good, contra) vs (bad, contra)	–1.19 (1.08)	1.59 (1.06)	–26.80	< .0001	[–2.99 –2.58]	–3.34
(good, contra) vs (good, ipsi)	–1.19 (1.08)	–0.92 (1.09)	–2.67	$= 7.7 \times 10^{-3}$	[–0.48 –0.07]	–0.33
(good, contra) vs (bad, ipsi)	–1.19 (1.08)	1.21 (1.13)	–23.12	< .0001	[–2.61 –2.20]	–2.89
(bad, contra) vs (good, ipsi)	1.59 (1.06)	–0.92 (1.09)	24.14	< .0001	[2.30 2.71]	3.01
(bad, contra) vs (bad, ipsi)	1.59 (1.06)	1.21 (1.13)	3.62	$= 3.0 \times 10^{-4}$	[0.17 0.58]	0.45
(good, ipsi) vs (bad, ipsi)	–0.92 (1.09)	1.21 (1.13)	–20.47	< .0001	[–2.33 –1.92]	–2.56
**Scene2**						
(good, contra) vs (bad, contra)	–1.12 (1.17)	1.76 (0.93)	–27.78	< .0001	[–3.08 –2.67]	–3.46
(good, contra) vs (good, ipsi)	–1.12 (1.17)	–0.78 (1.07)	–3.35	$= 8.0 \times 10^{-4}$	[–0.55 –0.14]	–0.42
(good, contra) vs (bad, ipsi)	–1.12 (1.17)	1.37 (1.05)	–24.01	< .0001	[–2.69 –2.29]	–2.99
(bad, contra) vs (good, ipsi)	1.76 (0.93)	–0.78 (1.07)	24.42	< .0001	[2.33 2.73]	3.04
(bad, contra) vs (bad, ipsi)	1.76 (0.93)	1.37 (1.05)	3.76	$= 2.0 \times 10^{-4}$	[0.19 0.59]	0.47
(good, ipsi) vs (bad, ipsi)	–0.78 (1.07)	1.37 (1.05)	–20.66	< .0001	[–2.34 –1.93]	–2.57
**Scene3**						
(good, contra) vs (bad, contra)	–1.41 (1.02)	1.57 (0.97)	–28.03	< .0001	[–3.18 –2.77]	–3.58
(good, contra) vs (good, ipsi)	–1.41 (1.02)	–1.01 (1.05)	–3.82	$= 1.0 \times 10^{-4}$	[–0.60 –0.19]	–0.48
(good, contra) vs (bad, ipsi)	–1.41 (1.02)	1.35 (0.96)	–26.67	< .0001	[–2.97 –2.56]	–3.32
(bad, contra) vs (good, ipsi)	1.57 (0.97)	–1.01 (1.05)	24.91	< .0001	[2.38 2.78]	3.10
(bad, contra) vs (bad, ipsi)	1.57 (0.97)	1.35 (0.96)	2.06	$= 4.0 \times 10^{-2}$	[0.01 0.42]	0.26
(good, ipsi) vs (bad, ipsi)	–1.01 (1.05)	1.35 (0.96)	–22.85	< .0001	[–2.57 –2.16]	–2.84
**Scene4**						
(good, contra) vs (bad, contra)	–1.25 (1.14)	1.66 (0.98)	–28.03	< .0001	[–3.11 –2.70]	–3.49
(good, contra) vs (good, ipsi)	–1.25 (1.14)	–0.92 (1.13)	–3.16	$= 1.6 \times 10^{-3}$	[-0.53 –0.12]	–0.39
(good, contra) vs (bad, ipsi)	–1.25 (1.14)	1.38 (1.05)	–25.30	< .0001	[–2.83 –2.42]	–3.15
(bad, contra) vs (good, ipsi)	1.66 (0.98)	–0.92 (1.13)	24.87	< .0001	[2.37 2.78]	3.10
(bad, contra) vs (bad, ipsi)	1.66 (0.98)	1.38 (1.05)	2.73	$= 6.4 \times 10^{-3}$	[0.08 0.49]	0.34
(good, ipsi) vs (bad, ipsi)	–0.92 (1.13)	1.38 (1.05)	–22.14	< .0001	[–2.50 –2.09]	–2.76

bioRxiv preprint doi: https://doi.org/10.1101/2024.12.25.630331; this version posted December 25, 2024. The copyright holder for this preprint (which was not certified by peer review) is the author/funder. This article is a US Government work. It is not subject to copyright under 17 USC 105 and is also made available for use under a CC0 license.

Table S4. Summary of statistical test to compare the normalized neuronal activity of SNr neurons at saccade onset among conditions during choice task in Figure S2.

SNr						
parametric bootstrap test (n = 10,000)	*p*					
full model vs. null model	< .0001					
post hoc (pairwise t-test, Bonferroni correction)	Mean (SD)	Mean (SD)	*t*	*p*	95% CI	effect size
**Scene1**						
(good, contra) vs (bad, contra)	–0.87 (1.17)	1.39 (1.21)	–20.16	< .0001	[–2.48 –2.04]	–2.52
(good, contra) vs (good, ipsi)	–0.87 (1.17)	–0.65 (1.71)	–1.97	= 4.9 $\times10^{-2}$	[–0.44 –0.01]	–0.25
(good, contra) vs (bad, ipsi)	–0.87 (1.17)	1.17 (1.25)	–18.16	< .0001	[–2.26 –1.82]	–2.27
(bad, contra) vs (good, ipsi)	1.39 (1.21)	–0.65 (1.71)	18.19	< .0001	[1.82 2.26]	2.27
(bad, contra) vs (bad, ipsi)	1.39 (1.21)	1.17 (1.25)	1.96	= 5.1 $\times10^{-2}$	[–0.01 0.44]	0.25
(good, ipsi) vs (bad, ipsi)	–0.65 (1.71)	1.17 (1.25)	–16.20	< .0001	[–2.04 –1.60]	–2.03
**Scene2**						
(good, contra) vs (bad, contra)	–0.80 (1.21)	1.54 (1.11)	–20.93	< .0001	[–2.56 –2.13]	–2.61
(good, contra) vs (good, ipsi)	–0.80 (1.21)	–0.54 (1.18)	–2.36	= 1.8 $\times10^{-2}$	[–0.48 –0.04]	–0.29
(good, contra) vs (bad, ipsi)	–0.80 (1.21)	1.39 (1.18)	–19.51	< .0001	[–2.41 –1.97]	–2.43
(bad, contra) vs (good, ipsi)	1.54 (1.11)	–0.70 (1.13)	18.57	< .0001	[1.86 2.30]	2.31
(bad, contra) vs (bad, ipsi)	1.54 (1.11)	1.39 (1.18)	1.38	= .167	[–0.07 0.38]	0.17
(good, ipsi) vs (bad, ipsi)	–0.70 (1.13)	1.39 (1.18)	–17.15	< .0001	[–2.15 –1.71]	–2.14
**Scene3**						
(good, contra) vs (bad, contra)	–1.11 (1.10)	1.35 (1.10)	–21.93	< .0001	[–2.68 –2.24]	–2.73
(good, contra) vs (good, ipsi)	–1.11 (1.10)	–0.80 (1.21)	–2.77	= 6.0 $\times10^{-3}$	[–0.53 –0.09]	–0.35
(good, contra) vs (bad, ipsi)	–1.11 (1.10)	1.26 (1.09)	–21.11	< .0001	[–2.59 –2.15]	–2.63
(bad, contra) vs (good, ipsi)	1.35 (1.10)	–0.80 (1.21)	19.16	< .0001	[1.93 2.37]	2.39
(bad, contra) vs (bad, ipsi)	1.35 (1.10)	1.26 (1.09)	0.82	= .410	[–0.13 0.31]	0.10
(good, ipsi) vs (bad, ipsi)	–0.80 (1.21)	1.26 (1.09)	–18.34	< .0001	[–2.27 –1.83]	–2.28
**Scene4**						
(good, contra) vs (bad, contra)	–0.87 (1.18)	1.43 (1.07)	–20.47	< .0001	[–2.51 –2.07]	–2.55
(good, contra) vs (good, ipsi)	–0.87 (1.18)	–0.68 (1.18)	–1.70	= 9.0 $\times10^{-2}$	[–0.41 –0.03]	–0.21
(good, contra) vs (bad, ipsi)	–0.87 (1.18)	1.30 (1.16)	–19.31	< .0001	[–2.38 –1.94]	–2.40
(bad, contra) vs (good, ipsi)	1.43 (1.07)	–0.68 (1.18)	18.78	< .0001	[1.88 2.32]	2.34
(bad, contra) vs (bad, ipsi)	1.43 (1.07)	1.30 (1.16)	1.17	= .244	[–0.09 0.35]	0.15
(good, ipsi) vs (bad, ipsi)	–0.68 (1.18)	1.30 (1.16)	–17.61	< .0001	[–2.19 –1.75]	–2.19

bioRxiv preprint doi: https://doi.org/10.1101/2024.12.25.630331; this version posted December 25, 2024. The copyright holder for this preprint (which was not certified by peer review) is the author/funder. This article is a US Government work. It is not subject to copyright under 17 USC 105 and is also made available for use under a CC0 license.

Table S5. Summary of statistical test to examine the relationship between the normalized neuronal activity and normalized saccade reaction times during choice task in Figure S2.

Condition	Mean (β) for NormalizedFR	95% Credible Interval for β	Probability β > 0	SD (Monkey)	SD (Neuron)	Residual SD	Rhat (β)	Effective Sample Size (β)
Contralateral Good	0.018	[-0.05, 0.08]	0.70	0.77	0.58	0.79	1.00	225328
Contralateral Bad	0.075	[0.01, 0.14]	0.99	0.27	0.82	0.59	1.00	197998
Ipsilateral Good	-0.043	[-0.11, 0.02]	0.11	0.54	0.67	0.73	1.00	202677
Ipsilateral Bad	0.010	[-0.05, 0.06]	0.57	0.33	0.84	0.56	1.00	144892

bioRxiv preprint doi: https://doi.org/10.1101/2024.12.25.630331; this version posted December 25, 2024. The copyright holder for this preprint (which was not certified by peer review) is the author/funder. This article is a US Government work. It is not subject to copyright under 17 USC 105 and is also made available for use under a CC0 license.

Table S6. Summary of statistical test to compare the normalized neuronal activity of SNr neurons among Return, Stay during choice task, and fixation task in Figure 3.

SNr						
parametric bootstrap test (n = 10,000)	*p*					
full model vs. null model	< .001					
post hoc<br>(pairwise t-test, Bonferroni correction)	Mean (SD)	Mean (SD)	*t*	*p*	95% CI	effect size
Contra						
(Return, choice) vs (Stay, choice)	1.59 (1.06)	1.40 (1.02)	1.83	$= 6.7 \times 10^{-2}$	[–0.01 0.42]	0.25
(Return, choice) vs (Good, fixation)	1.59 (1.06)	1.31 (1.06)	–2.71	$= 6.8 \times 10^{-3}$	[–0.51 –0.08]	–0.37
(Return, choice) vs (Bad, fixation)	1.59 (1.06)	1.57 (0.84)	–0.29	$= 7.7 \times 10^{-1}$	[–0.24 0.18]	–0.04
(Stay, choice) vs (Good, fixation)	1.40 (1.02)	1.31 (1.06)	–0.78	$= 4.3 \times 10^{-1}$	[–0.32 0.14]	–0.11
(Stay, choice) vs (Bad, fixation)	1.40 (1.02)	1.57 (0.84)	1.44	$= 1.5 \times 10^{-1}$	[–0.06 0.40]	0.21
(Good, fixation) vs (Bad, fixation)	1.31 (1.06)	1.57 (0.84)	2.30	$= 2.1 \times 10^{-2}$	[0.04 0.48]	0.33
Ipsi						
(Return, choice) vs (Stay, choice)	1.21 (1.13)	1.17 (0.87)	0.76	$= 7.6 \times 10^{-1}$	[–0.18 0.25]	0.04
(Return, choice) vs (Good, fixation)	1.21 (1.13)	0.90 (0.82)	–3.05	$= 2.3 \times 10^{-3}$	[–0.54 –0.12]	–0.42
(Return, choice) vs (Bad, fixation)	1.21 (1.13)	0.51 (0.85)	–6.65	< .0001	[–0.93 –0.51]	–0.90
(Stay, choice) vs (Good, fixation)	1.17 (0.87)	0.90 (0.82)	–2.51	$= 1.2 \times 10^{-2}$	[–0.53 –0.06]	–0.37
(Stay, choice) vs (Bad, fixation)	1.17 (0.87)	0.51 (0.85)	–5.80	< .0001	[–0.92 –0.45]	–0.86
(Good, fixation) vs (Bad, fixation)	0.90 (0.82)	0.51 (0.85)	–3.44	$= 6.0 \times 10^{-4}$	[–0.61 –0.17]	–0.49

bioRxiv preprint doi: https://doi.org/10.1101/2024.12.25.630331; this version posted December 25, 2024. The copyright holder for this preprint (which was not certified by peer review) is the author/funder. This article is a US Government work. It is not subject to copyright under 17 USC 105 and is also made available for use under a CC0 license.

Table S7. Summary of statistical test to compare the effects of CPP + NBQX injection into SNr during choice task in Figure 4.

Injection during choice task				
parametric bootstrap test (n = 10,000)	*p*			
full model vs. null model	< .0001			
post hoc<br>(pairwise t-test, Bonferroni correction)	Mean (ms)<br>(SD)	Mean (ms)<br>(SD)	*z*	*p*
CPP+NBQX Contra Good pre vs. post	196.25<br>(9.11)	171.42<br>(12.17)	–4.48	< .0001
Saline Contra Good pre vs. post	194.17<br>(8.68)	191.08<br>(7.81)	–0.54	= .58
CPP+NBQX Contra Bad pre vs. post	267.75<br>(18.52)	181.42<br>(17.83)	–14.01	< .0001
Saline Contra Bad pre vs. post	271.42<br>(17.73)	264.42<br>(22.38)	–1.05	= .29
CPP+NBQX Ipsi Good pre vs. post	199.25<br>(10.17)	214.92<br>(22.70)	2.67	= 7.7 $\times 10^{-3}$
Saline Ipsi Good pre vs. post	199.83<br>(8.83)	195.92<br>(8.27)	–0.68	= .50
CPP+NBQX Ipsi Bad pre vs. post	282.58<br>(31.80)	280.08<br>(58.68)	–0.37	= .72
Saline Ipsi Bad pre vs. post	288.75<br>(36.97)	274.83<br>(38.89)	–2.03	= 4.2 $\times 10^{-2}$

bioRxiv preprint doi: https://doi.org/10.1101/2024.12.25.630331; this version posted December 25, 2024. The copyright holder for this preprint (which was not certified by peer review) is the author/funder. This article is a US Government work. It is not subject to copyright under 17 USC 105 and is also made available for use under a CC0 license.

Table S8. Summary of statistical test to compare the effects of CPP + NBQX injection into SNr while monkeys chose actions for Bad object during Choice task in Figure 4.

Injection into SNr during Choice task				
Accept Bad object				
parametric bootstrap test (n = 10,000)	*p*			
full model vs. null model	= 1.00			
Return for Bad object				
parametric bootstrap test (n = 10,000)	*p*			
full model vs. null model	< .001			
post hoc (pairwise t-test, Bonferroni correction)	Mean (%) (SD)	Mean (%) (SD)	*z*	*p*
CPP+NBQX Return Contra Bad pre vs. post	80.94 (15.30)	88.73 (10.99)	3.41	$= 7.0 \times 10^{-4}$
Saline Return Contra Good pre vs. post	78.93 (13.43)	77.99 (14.15)	–0.63	= .53
CPP+NBQX Return Ipsi Bad pre vs. post	72.04 (20.47)	48.38 (24.41)	–8.14	< .0001
Saline Return Ipsi Bad pre vs. post	68.13 (17.52)	70.48 (19.81)	0.28	= .78
Stay for Bad object				
parametric bootstrap test (n = 10,000)	*p*			
full model vs. null model	< .001			
post hoc (pairwise t-test, Bonferroni correction)	Mean (%) (SD)	Mean (%) (S.D.)	*z*	*p*
CPP+NBQX Return Contra Bad pre vs. post	19.51 (15.31)	3.70 (8.61)	–6.47	< .0001
Saline Return Contra Good pre vs. post	21.07 (13.42)	21.68 (13.82)	0.38	= .70
CPP+NBQX Return Ipsi Bad pre vs. post	27.78 (20.47)	50.46 (23.36)	7.90	< .0001
Saline Return Ipsi Bad pre vs. post	31.79 (17.55)	29.37 (19.58)	–0.40	= .69

bioRxiv preprint doi: https://doi.org/10.1101/2024.12.25.630331; this version posted December 25, 2024. The copyright holder for this preprint (which was not certified by peer review) is the author/funder. This article is a US Government work. It is not subject to copyright under 17 USC 105 and is also made available for use under a CC0 license.

Table S9. Summary of statistical test to compare the effects of CPP + NBQX injection into *SNr during fixation task in Figure 4.*

Injection into SNr during fixation task				
parametric bootstrap test (n = 10,000)	*p*			
full model vs. null model	< .05			
post hoc<br>(pairwise t-test, Bonferroni correction)	Mean (%)<br>(SD)	Mean (%)<br>(SD)	*t*	*z*
CPP+NBQX Contra Good pre vs. post	2.62<br>(4.23)	63.97<br>(25.43)	14.85	< .0001
Saline Contra Good pre vs. post	1.85<br>(2.12)	2.57<br>(3.40)	0.65	= .51
CPP+NBQX Contra Bad pre vs. post	1.86<br>(2.47)	52.51<br>(24.40)	12.10	< .0001
Saline Contra Bad pre vs. post	0.93<br>(1.89)	0.75<br>(1.54)	–0.08	= .94
CPP+NBQX Ipsi Good pre vs. post	2.43<br>(3.32)	0.17<br>(0.58)	–2.18	= .03
Saline Ipsi Good pre vs. post	0.51<br>(0.97)	0.67<br>(1.21)	0.21	= .84
CPP+NBQX Ipsi Bad pre vs. post	1.22<br>(2.50)	0.80<br>(1.19)	–0.79	= .43
Saline Ipsi Bad pre vs. post	0.24<br>(0.82)	0.22<br>(0.76)	0.06	= .95
